# Inhibition of Antimicrobial-Resistant *Escherichia coli* Using a Broad Host Range Phage Cocktail Targeting Various Bacterial Phylogenetic Groups

**DOI:** 10.3389/fmicb.2021.699630

**Published:** 2021-08-25

**Authors:** Jinshil Kim, Haejoon Park, Sangryeol Ryu, Byeonghwa Jeon

**Affiliations:** ^1^Department of Food and Animal Biotechnology, Department of Agricultural Biotechnology, and Research Institute for Agriculture and Life Sciences, Seoul National University, Seoul, South Korea; ^2^Center for Food Bioconvergence, Seoul National University, Seoul, South Korea; ^3^Divison of Environmental Health Sciences, School of Public Health, University of Minnesota, Minneapolis, MN, United States

**Keywords:** bacteriophage, cocktail, antimicrobial-resistant *Escherichia coli*, raw chicken, phage cocktail

## Abstract

Antimicrobial-resistant (AMR) commensal *Escherichia coli* is a major reservoir that disseminates antimicrobial resistance to humans through the consumption of contaminated foods, such as retail poultry products. This study aimed to control AMR *E. coli* on retail chicken using a broad host range phage cocktail. Five phages (JEP1, 4, 6, 7, and 8) were isolated and used to construct a phage cocktail after testing infectivity on 67 AMR *E. coli* strains isolated from retail chicken. Transmission electron microscopic analysis revealed that the five phages belong to the *Myoviridae* family. The phage genomes had various sizes ranging from 39 to 170 kb and did not possess any genes associated with antimicrobial resistance and virulence. Interestingly, each phage exhibited different levels of infection against AMR *E. coli* strains depending on the bacterial phylogenetic group. A phage cocktail consisting of the five phages was able to infect AMR *E. coli* in various phylogenetic groups and inhibited 91.0% (61/67) of AMR *E. coli* strains used in this study. Furthermore, the phage cocktail was effective in inhibiting *E. coli* on chicken at refrigeration temperatures. The treatment of artificially contaminated raw chicken skin with the phage cocktail rapidly reduced the viable counts of AMR *E. coli* by approximately 3 log units within 3 h, and the reduction was maintained throughout the experiment without developing resistance to phage infection. These results suggest that phages can be used as a biocontrol agent to inhibit AMR commensal *E. coli* on raw chicken.

## Introduction

*Escherichia coli* is the most common enteric bacteria inhabiting the gastrointestinal tract of a wide range of animals and humans (Kaper et al., 2004). Due to the ubiquitousness in the intestines, commensal *E. coli* is likely to be exposed to orally ingested antibiotics and develops antimicrobial resistance in food-producing animals and may act as a donor and a recipient of antimicrobial resistance genes (Poirel et al., 2018). Antimicrobial-resistant (AMR) commensal *E. coli* is frequently isolated from food-producing animals and their meat products (Szmolka and Nagy, 2013). Although commensal *E. coli* does not cause AMR infection in humans, AMR commensal *E. coli* is considered a major reservoir for disseminating antimicrobial resistance to humans. For instance, extended-spectrum β-lactamases (ESBL)-producing *E. coli* is highly prevalent in retail poultry (Saliu et al., 2017). ESBL are β-lactamase enzymes conferring resistance to all β-lactam drugs except carbapenem and mainly located on conjugative plasmids, enhancing their rapid spread in *E. coli* populations and pathogenic bacterial species in the Enterobacterales order (Rupp and Fey, 2003).

AMR bacteria in food-producing animals can be transmitted to foods during processing and subsequently to humans through the consumption of contaminated foods. According to the Centers for Disease Control and Prevention (CDC), approximately 1 in 5 AMR infections in the United States are associated with food and animals (CDC, 2013). Since meat and poultry are the major food commodity implicated in 35% of foodborne illnesses in the United States (Painter et al., 2013; Dewey-Mattia et al., 2018), antimicrobial resistance originating from food-producing animals poses a serious public health concern. Particularly, AMR bacteria are frequently isolated from retail raw chickens (do Monte et al., 2017; Schrauwen et al., 2017; Wang et al., 2017). Our previous studies also showed that ESBL-producing *E. coli* is highly prevalent in retail poultry (Park et al., 2019). In addition, we isolated from retail chicken a pan drug-resistant *E. coli* possessing a plasmid harboring *mcr-1*, which confers resistance to colistin, one of the last resort antibiotics to treat Gram-negative infections (Liu et al., 2016; Kim et al., 2019).

To mitigate the public health risk of antimicrobial resistance, it is important to control the sources that are involved in the spread of antimicrobial resistance. Especially, AMR commensal *E. coli* in chickens is an important target to control because it is highly prevalent and capable of transferring antimicrobial resistance to pathogenic bacteria, such as pathogenic *E. coli* and *Salmonella* (Nhung et al., 2017). Among non-antibiotic-based intervention measures for the control of AMR commensal *E. coli*, bacteriophages (phages) are considered an ideal antimicrobial alternative because phages specifically infect only target bacteria with completely different antimicrobial mechanisms from those of existing antibiotics (Sulakvelidze et al., 2001; Skurnik and Strauch, 2006; Endersen et al., 2014). Whereas antibiotics affect bacteria other than pathogens, moreover, phages can selectively infect only the target bacteria (Altamirano and Barr, 2019; Nogueira et al., 2019). However, the strict host specificity of phage infection is rather a disadvantage because the host range is generally too narrow to inhibit bacteria with wide genetic diversity (Bert et al., 2010; Talukdar et al., 2013; Park et al., 2019). To overcome the limitations, phages are generally used in a cocktail by mixing phages capable of infecting a range of different hosts (Nilsson, 2014). In this study, we isolated phages that preferentially infect the major phylogenetic groups of *E. coli* isolates from retail chickens and developed a phage cocktail that effectively inhibited AMR *E. coli* on chicken carcasses.

## Materials and Methods

### Phage Isolation and Stock Preparation

Sixty-seven AMR *E. coli* strains (E1–E67) were isolated from retail raw chicken in our previous study (Park et al., 2019). The AMR *E. coli* strains and *E. coli* MG1655 were routinely cultured at 37°C in Luria-Bertani (LB) media (Difco, United States). Phages were isolated from food (retail chicken and duck carcasses), sewage, and animal (chicken and pig) feces as described previously (Kim and Ryu, 2011). Briefly, the samples were homogenized by vortexing in sodium chloride-magnesium sulfate (SM) buffer (100 mM NaCl, 8 mM MgSO_4_^.^7H_2_O, and 50 mM Tris^.^HCl, pH 7.5). After centrifugation at 10,000 × *g* for 5 min, the supernatant was filtered through a 0.22 μm pore sized filter (Millipore, United States). Five milliliters of filtered samples were mixed with the equal volume of 2 × LB broth and 100 μl overnight culture of the AMR *E. coli* strains. After incubation at 37°C overnight, the culture was centrifuged and filter-sterilized. To confirm the presence of phages, supernatants were serially diluted and spotted on 0.4% LB soft top agar containing an overnight culture of AMR *E. coli* strains. After incubation at 37°C overnight, a single plaque was picked and eluted with 1 ml SM buffer. This step was repeated at least three times for each plaque.

To propagate phages, the incubation time was determined based on the lysis activity of each phage. The purified lysate was added to the culture of exponentially grown propagation host strains (JEP1: *E. coli* MG1655, JEP4: *E. coli* E15, JEP6: *E. coli* E55), and the mixture was incubated at 37°C for 4 h (JEP1, 4, and 6) in LB broth. Also, the purified lysates of JEP7 and JEP8 were incubated with the overnight culture of propagation host strains (JEP7: *E. coli* E61, JEP8: *E. coli* E63) overnight in LB broth. Phage propagation was performed with three different culture volumes (4, 40, and 250 ml LB broth), and then the culture was centrifuged and filtered. Phage particles were precipitated by mixing with polyethylene glycol (PEG) 6000 (Junsei Chemical Co. Ltd., Japan) and 1 M NaCl. Finally, CsCl density gradient ultracentrifugation (Himac CP 100b, Hitachi, Japan) with CsCl step densities (1.3, 1.45, 1.5, and 1.7 g/ml) at 78,500 × *g* was conducted at 4°C for 2 h. After centrifugation, a blue band of viral particles was collected and dialyzed twice for 1 h in 1 L of SM buffer. The concentrated phage stocks were stored at 4°C until used.

### Determination of Phage Host Range

A total of 67 strains of AMR *E. coli* were used to assess the host ranges of eight phage infections. Each strain was incubated at 37°C overnight with shaking (200 rpm), and then 100 μl of each bacterial culture was added to 5 ml of 0.4% LB soft agar and mixed. The mixture was overlaid on a 1.5% LB agar plate and dried at room temperature for 20 min. Subsequently, 10-fold serially diluted by SM buffer of each phage lysates were spotted onto a prepared bacterial lawn and incubated at 37°C for 12 h. After incubation, the formation of single plaques was recorded to determine the phage sensitivity of each strain. The efficiency of phage infection of each strain was compared to that of the host strain.

### Transmission Electron Microscopy Analysis

The CsCl-purified phages were morphologically characterized with transmission electron microscopy (TEM) analysis. Briefly, 10 μl of purified phage (ca. 1 × 10^10^ PFU/ml) was placed on carbon-coated formvar/copper grids (200 mesh) and negatively stained with 2% aqueous uranyl acetate (pH 4.0) for 10 s. The phages were observed with energy-filtering TEM (LIBRA 120, Carl Zeiss, Germany) at 120-kV accelerating voltage at the National Instrumentation Center for Environmental Management (Seoul, South Korea). The phages were identified and classified using the International Committee on Taxonomy of Viruses (ICTV) classification (King et al., 2011).

### DNA Purification and Whole-Genome Sequencing of Phages

To extract genomic DNA from phages, bacterial nucleic acids were removed by DNase I and RNase A (1 μg/ml each) at room temperature for 30 min. The virions were then lysed by incubating with a mixture [final concentration of 50 μg/ml proteinase K, 20 mM ethylenediaminetetraacetic acid (EDTA), 0.5% sodium dodecyl sulfate (SDS)] at 56°C for 1 h. After lysis, DNA was purified by phenol-chloroform extraction and precipitated by ethanol. The library was constructed with the Illumina TruSeq DNA library prep kit using purified genomic phage DNA. It was sequenced using the Illumina Miseq platform (300 bp paired ended) and assembled with GS de novo assembler software (Roche, Switzerland) at Sanigen Inc., South Korea. Prediction of open reading frames (ORFs) was performed using the combination of Glimmer3 and GeneMarkS2 software. The complete genome sequences of JEP1, 4, 6, 7, and 8 were deposited in GenBank with the accession numbers of MT740314, MT740315, MT764206, MT764207, and MT764208, respectively. The presence of genes associated with antimicrobial resistance and virulence in the phage genomes was examined with ResFinder 4.1^1^ and VirulenceFider 2.0^2^, respectively.

### Phylogenetic Analysis of Phages

Phylogenetic analysis of the five phages was performed in comparison with sixty *E. coli* phages in the *Myoviridae* family, which were reported in a previous study (Korf et al., 2019) using VICTOR^3^ that performs based on genome-BLAST Distance phylogeny (GBDP) method. The phage sequences were obtained through the NCBI nucleotide databases^4^. All pairwise comparisons of the amino acid sequences were conducted using the GBDP method (Meier-Kolthoff et al., 2013) under the settings recommended for prokaryotic viruses (Meier-Kolthoff and Göker, 2017). The resulting intergenomic distances were used to infer a balanced minimum evolution tree with branch support via FASTME including SPR postprocessing for formulas D4 (Lefort et al., 2015). The branch support was inferred from 100 pseudo-bootstrap replicates each. Trees were rooted at the midpoint (Farris, 1972) and visualized with FigTree (Rambaut, 2006). The taxon boundaries at the species, genus, and family levels were estimated with the OPTSIL program (Göker et al., 2009) using the recommended clustering thresholds (Meier-Kolthoff and Göker, 2017) and an F value (fraction of links required for cluster fusion) of 0.5 (Meier-Kolthoff et al., 2014).

### Phage Inhibition Assays

The infection efficiency of the phage cocktail was evaluated with mixed cultures of *E. coli* strains which were randomly selected from the major phylogenetic groups (A,B1,B2, and D), including mixed culture 1 (E20, E41, E55, and E59), mixed culture 2 (E3, E43, E55, and E59), mixed culture 3 (E17, E41, E52, and E59), and mixed culture 4 (E20, E45, E52, and E59). The each strain was incubated at 37°C with shaking (200 rpm) overnight. The mixed culture of the *E. coli* strains was prepared by transferring 1% (v/v) of each strain to fresh LB broth. Then, a single phage or the phage cocktail was added to the bacterial suspension at a multiplicity of infection (MOI) of 1. The optical density at 600 nm (OD_600_) was measured with the SpectraMax i3 multimode microplate reader (Molecular Devices, Sunnyvale, CA, United States) for 12 h. The infection assay was also performed at 4 and 25°C. After cultivation to an OD_600_ of 0.5, the mixed culture of AMR *E. coli* strains (E20, E41, E55, and E59) was diluted in LB broth and added to 4 ml of LB broth at 10^5^ CFU/ml. The infection was initiated by adding the 10 μl phage cocktail (approximately 10^8^ PFU/ml; MOI 10^3^), and the SM buffer was used as a control. The cultures were incubated with shaking (200 rpm) at food storage and handling temperatures (4 and 25°C), and samples were taken at 3, 6, and 24 h postinfection for bacterial counting. Viable counts were determined by 10-fold serial dilution in PBS and plating on LB agar plates.

### Inhibition of AMR *E. coli* on Raw Chicken Skin by Phage Cocktail

Raw chicken skin samples were purchased from retail stores, and the skin was cut into a 2 cm × 2 cm square with a sterilized razor. For decontamination, the chicken skin samples were immersed in 70% ethanol overnight and UV-treated on both sides for 30 min in a biosafety cabinet. Mixed culture 1 (E20, E41, E55, and E59), which was used in the phage inhibition assay, was prepared as mentioned above and diluted to 8 × 10^6^ CFU/ml in PBS. Then 50 μl of the mixed culture of the *E. coli* strains was spotted onto a 2 cm × 2 cm chicken skin to achieve the final inoculum level of approximately 5 log CFU/cm^2^ on a chicken skin sample. The same volume of PBS was added as a negative control. Samples were dried in a biosafety cabinet for 30 min. Then 100 μl of the phage cocktail (MOI = 10^3^) or SM buffer (control) were spotted onto chicken skin samples and incubated at 4 and 25°C. At 3, 6, and 12 h of incubation, each sample was mixed with 10 ml 0.1% buffered peptone water (BPW) and vortexed for 2 min in a 50 ml tube. After removal of the chicken skin, the mixture was centrifuged at 10,000 × *g* for 5 min, and pellets were resuspended with 10 ml of BPW. Viable counts were determined by 10-fold serial dilution in PBS and plating on LB agar plates.

## Results

### Determination of the Host Range of Phages Infecting AMR *E. coli* Isolates From Raw Chicken

**FIGURE 1:**
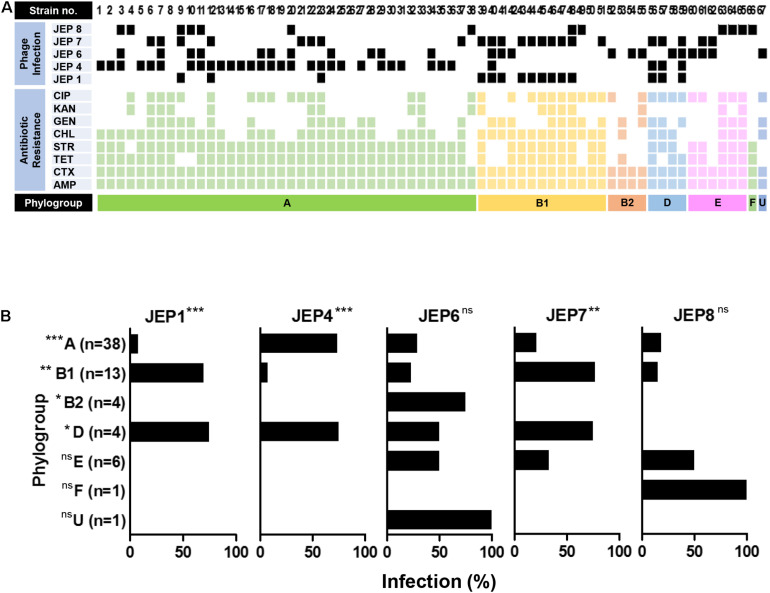
Host range of the five phages. **(A)** The phage infection of 67 strains of AMR *E. coli* isolated from retail chicken. The antimicrobial resistance patterns of the *E. coli* strains and the infectivity of five phages are indicated. CIP, ciprofloxacin; KAN, kanamycin; GEN, gentamicin; CHL, chloramphenicol; STR, streptomycin; TET, tetracycline; CTX, cefotaxime; AMP, ampicillin; and phylogroup U, Unknown. **(B)** Association of the phylogenetic groups of AMR *E. coli* with the infection frequency of the five phages. The experiment was repeated three times. Statistical analysis was performed using the chi-square test with GraphPad Prism (*P < 0.05; **P < 0.01; ***P < 0.001; ns, not significant).

A total of eight *E. coli* phages were isolated from meat, sewage, and animal fecal samples (Table 1 and Supplementary Table 1) and used in phage infection assays with 67 AMR *E. coli* isolates from retail chicken (Figure 1A and Supplementary Tables 2, 3). Among the eight *E. coli* phages, five phages (JEP1, 4, 6, 7, and 8) were selected for the construction of a cocktail mainly because of their host range (Figure 1A and Table 2). Importantly, the design to construct a phage cocktail was mainly based on the differential infection frequencies depending on the phylogenetic group of *E. coli* (Figure 1B). For instance, JEP4 phage infected 73.7% (28/38) of AMR *E. coli* strains in phylogroup A, whereas JEP1 and JEP7 phages effectively infected the strains in phylogroups B1 [69.3% (9/13) and 76.9% (10/13), respectively] (Figure 1 and Table 2). The three phages (JEP1, JEP4, and JEP7) showed similar inhibition frequencies against *E. coli* strains in phylogenetic group D (Figure 1 and Table 2). A cocktail was constructed to target various phylogenetic groups of *E. coli* based on the infection pattern dependent on the phylogenetic group. *E. coli* isolates in phylogenetic groups A, B1, B2, and D could be inhibited by JEP4, JEP1 & 7, JEP6, and JEP1, 4 & 7, respectively (Figure 1B and Table 2). In addition, JEP8 was included in the cocktail to inhibit *E. coli* isolates in the minor phylogenetic groups (i.e., E and F) (Figure 1B and Table 2). The cocktail consisting of the five phages infected 91.0% (61/67) of AMR *E. coli* strains isolated from retail chicken (Figure 1 and Table 2).

**TABLE 1:** Morphological and genomic features of the five phages used in the cocktail.

Phage	Isolation source	Morphological features (nm; n = 3)		Genomic features				
			
		Head	Tail	Size (bp)	GC (%)	ORF^a^	tRNA	Accession No.
JEP1	Retail chicken	79.6 ± 1.9	101.0 ± 3.8	143,610	43.54	223	5	MT740314
JEP4	Chicken feces	106.3 ± 5.5	102.9 ± 2.6	39,195	47.05	61	0	MT740315
JEP6	Pig feces	109.1 ± 1.7	110.3 ± 2.3	170,340	35.31	274	7	MT764206
JEP7	Retail duck	103.9 ± 5.2	95.22.4	52,936	45.94	71	0	MT764207
JEP8	Retail chicken	96.1 ± 2.5	95.9 ± 3.5	165,295	40.47	272	0	MT764208

*^*a*^Open reading frame.*

**TABLE 2:** Infection frequencies of the five phages used in the cocktail.

Phage	Phylogenetic group of ESBL-producing *E. coli*							Total (*n* = 67)
	A (*n* = 38)	B1 (*n* = 13)	B2 (*n* = 4)	D (*n* = 4)	E (*n* = 6)	F (*n* = 1)	U^a^ (*n* = 1)	
JEP1	3(7.9%)	9(69.3%)	0(0.0%)	3(75.0%)	0(0.0%)	0(0.0%)	0(0.0%)	15(22.4%)
JEP4	28(73.7%)	1(7.7%)	0(0.0%)	3(75.0%)	0(0.0%)	0(0.0%)	0(0.0%)	32(47.8%)
JEP6	11(28.9%)	3(23.1%)	3(75.0%)	2(50.0%)	3(50.0%)	0(0.0%)	1(100.0%)	23(34.3%)
JEP7	8(21.1%)	10(76.9%)	0(0.0%)	3(75.0%)	2(33.3%)	0(0.0%)	0(0.0%)	23(34.3%)
JEP8	7(18.4%)	2(15.4%)	0(0.0%)	0(0.0%)	3(50.0%)	1(100.0%)	0(0.0%)	13(19.4%)
Total	34(89.5%)	12(92.3%)	3(75.0%)	4(100.0%)	6(100.0%)	1(100.0%)	1(100.0%)	61(91.0%)

*^a^unknown.*

### Characterization of the Five *E. coli* Phages

**FIGURE 2:**
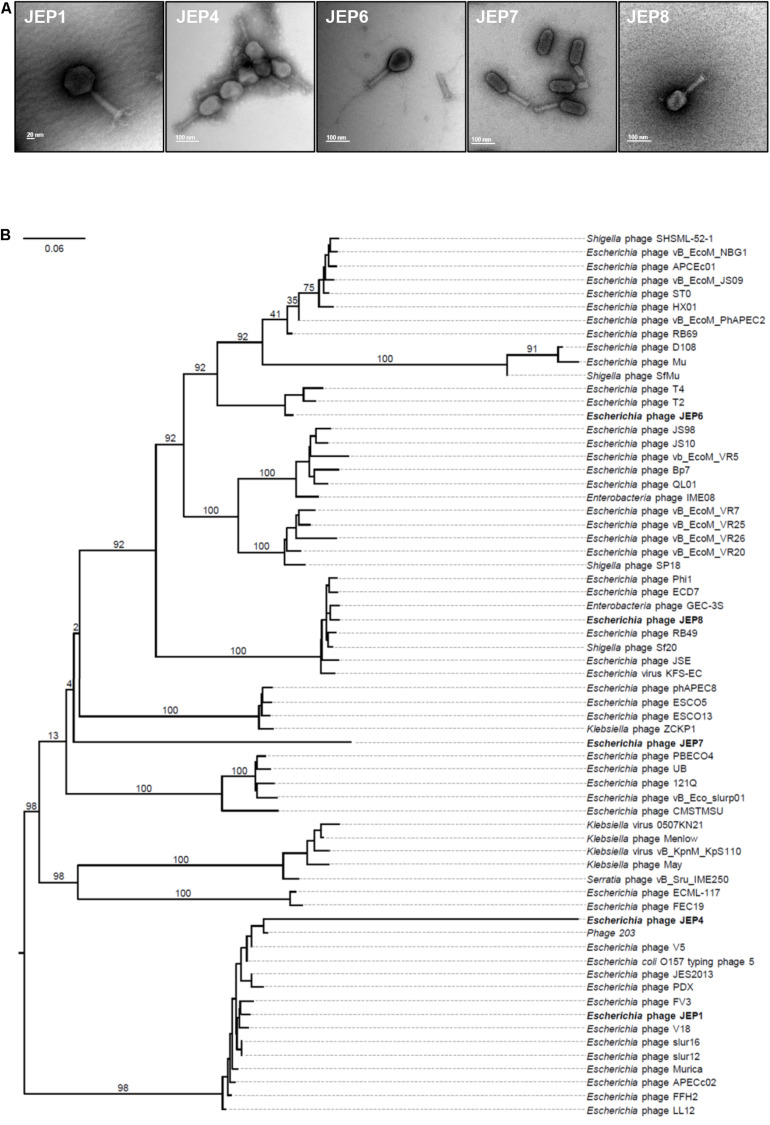
The morphological and genomic features of phages. **(A)** Transmission electron microscopy (TEM) images of the five phages. **(B)** Phylogenetic analysis showing the relationship between the five phages and 60 *Myoviridae* family phages that were reported in a previous study (Korf et al., 2019).

The morphology and genome sequences of JEP1, 4, 6, 7, and 8 phages were analyzed. Based on the TEM analysis, the five phages exhibited the typical morphological features of the *Myoviridae* family, such as a big head and an inflexible/contractile tail (Figure 2A and Table 1). The phages had various genome sizes ranging from 39 kb (JEP4) to 170 kb (JEP6) (Table 1), and genes associated with antimicrobial resistance and virulence were not found in the phage genomes (data not shown). The phylogenetic association of the five phages was analyzed with previously reported *E. coli* phages in the *Myoviridae* family (Korf et al., 2019). JEP1 & JEP4 and JEP6 & JEP8 belonged to the same genus clusters, and JEP7 belonged to a different genus cluster (Figure 2B). At the species level, the five phages were clustered into different groups, indicating that the five phages are phylogenetically distinct from each other.

### Inhibition of AMR *E. coli* With the Phage Cocktail

**FIGURE 3:**
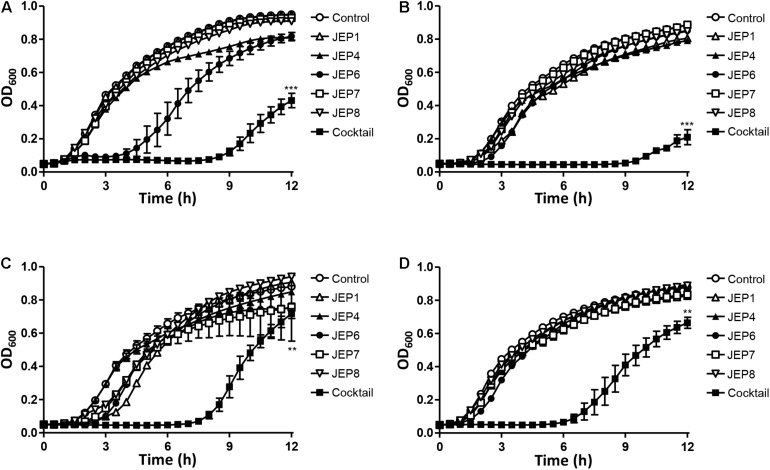
Phage inhibition of mixed cultures of AMR *E. coli* strains in LB broth at 37°C. The four mixed cultures used in the assay included: **(A)** Mixed culture 1 consisting of AMR *E. coli* strains E20, E41, E55, and E59, **(B)** Mixed culture 2 consisitng of E3, E43, E55, and E59, **(C)** Mixed culture 3 consisiting of E17, E41, E52, and E59, and **(D)** Mixed culture 4 consisting of E20, E45, E52, and E59. The reduction in the OD_600_ of the mixed culture of AMR *E. coli* strains was measured after treatment with single phages or the phage cocktail. The data present the means and the standard errors of the mean (SEM) of the results of three experiments. Statistical analysis was performed using a Student’s *t*-test compared to the control in the same sampling (12 h) with GraphPad Prism (**P < 0.01; ***P < 0.001).

**FIGURE 4:**
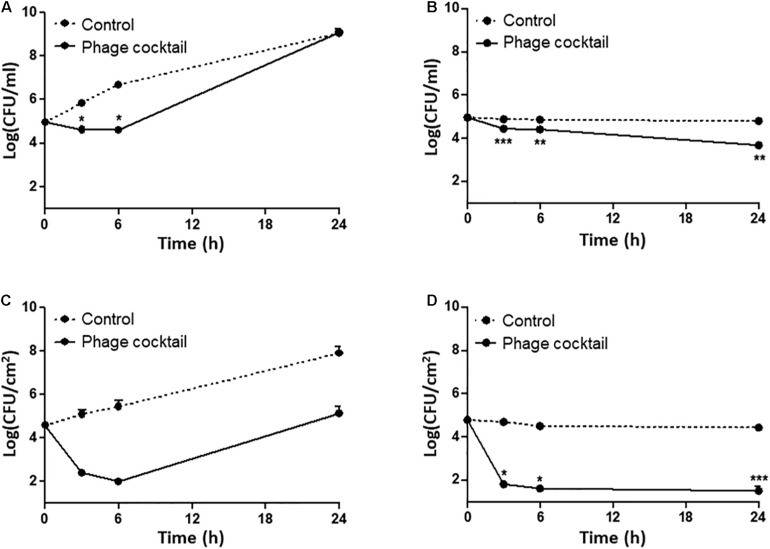
Phage inhibition of the viability of a mixed culture of AMR *E. coli* isolates (E20, E41, E55, and E59) in LB broth **(A,B)** and on raw chicken skin **(C,D)**. The viable counts of a mixed culture of AMR *E. coli* strains in LB broth without (control) and with the phage cocktail at 25°C **(A)** and 4°C **(B)**. The levels of AMR *E. coli* isolates on chicken skin without (control) and with the phage cocktail at 25°C **(C)** and 4°C **(D)**. The data show the means and the standard errors of the mean (SEM) of the results of three experiments. Statistical analysis was performed using a Student’s *t*-test compared to the non-treated control with GraphPad Prism (*P < 0.05; **P < 0.01; ***P < 0.001).

Mixed cultures of AMR *E. coli* strains were treated with the phage cocktail to evaluate infection efficiency because retail raw chicken is normally contaminated by multiple strains, not a single. *E. coli* strains were randomly selected from the major phylogenetic groups A, B1, B2, and D, combined in a single culture, and treated with each single phage or the phage cocktail. The treatment of mixed cultures with single phages did not, or only marginally, reduce the growth of mixed cultures of *E. coli*. However, the phage cocktail substantially inhibited the growth of the mixed cultures (Figure 3). In mixed cultures 2 (Figures 3B) and 4 (Figure 3D), for instance, single phages did not cause any growth reduction compared to the non-treated negative control, whereas the phage cocktail markedly reduced the growth of *E. coli* strains in mixed cultures. These results indicated the phage cocktail was effective at simultaneously inhibiting multiple strains of *E. coli* belonging to different phylogenetic groups.

Because raw chicken products are preserved normally at refrigeration temperatures and sometimes exposed to room temperatures during handling, we evaluated the infection frequency at 4 and 25°C. At 25°C, the treatment with the phage cocktail significantly reduced the level of AMR *E. coli* strains at the beginning of infection (approximately 0.35 log CFU/ml after 6 h; *P* = 0.0378), but further incubation did not make a difference in the viable counts of *E. coli* compared to the non-treated control (Figure 4A). However, the level of AMR *E. coli* was significantly reduced at 4°C within a few hours, and the reduction was maintained during the entire course of the experiment (Figure 4B). We examined the inhibition efficiency of the phage cocktail on raw chicken skin. To mimic the situation of food contamination, raw chicken skin was artificially contaminated with the mixed culture of AMR *E. coli* strains. Compared to LB media (Figures 4A,B), interestingly, the phage cocktail reduced AMR *E. coli* more significantly on chicken skin at both 4 and 25°C (Figures 4C,D). When raw chicken skin samples were treated with the phage cocktail at an MOI of 10^3^ at 25°C, the mixed culture of AMR *E. coli* strains was rapidly reduced by 2.19 log CFU/cm^2^ and 2.58 log CFU/cm^2^ after 3 h and 6 h, respectively (Figure 4C). After that, the mixed culture of AMR *E. coli* strains continued to grow on chicken skin (Figure 4C). At 4°C, however, the treatment of raw chicken skin with the phage cocktail significantly reduced the level of AMR *E. coli* strains within 3 h and continued to reduce the viable counts of AMR *E. coli* by 3.17 CFU/cm^2^ and 3.28 log CFU/cm^2^ after 6 and 24 h, respectively (Figure 4D). The results showed that the phage cocktail is highly effective in inhibiting AMR *E. coli* on chicken carcasses especially at refrigeration temperatures.

## Discussion

Since bacteria develop phage resistance rapidly (Labrie et al., 2010), phages are normally treated in a cocktail using those that recognize different host receptors (Tanji et al., 2004; Gu et al., 2012; Yen et al., 2017). This is because, if bacteria develop resistance to one phage, another phage in the cocktail, which recognizes a different receptor, still can infect the bacteria. However, the identification of the host receptor of a phage requires a series of experiments involving mutagenesis, which is often labor-intensive and time-consuming. Without identifying the host receptors of *E. coli* phages, in this study, we constructed a phage cocktail that can effectively infect *E. coli* isolates from retail chicken using phages that preferentially infect the major phylogenetic groups of *E. coli*. The Clermont phylotyping classifies *E. coli* into four major (A,B1,B2, and D) and two minor groups (E and F) (Clermont et al., 2013). The phylogenetic group of *E. coli* is related to certain pathotypes and the host origin. For instance, phylogroups A and B2 normally predominate in human strains, while *E. coli* isolates from chicken mostly belong to phylogroups A and B1 (Unno et al., 2009; Kluytmans et al., 2012; Xu et al., 2014; Coura et al., 2015). Consistently, in our previous study, AMR *E. coli* strains isolated from retail chicken dominantly belonged to phylogroups A and B1 (Park et al., 2019). In the cocktail, we included three phages (JEP1, JEP4, and JEP7) that infected the major phylogenetic groups of *E. coli* isolates from chicken (i.e., A and B1) (Figure 1B and Table 2), and the phage cocktail infected 91.0% of the tested AMR *E. coli* strains (Figure 1A and Table 2). The same approach can be used to construct phage cocktails to target other pathogenic bacteria demonstrating unique phylogenetic features. For example, extraintestinal pathogenic *E. coli* (ExPEC) predominates phylogroups B2 and D (Picard et al., 1999; Cortés et al., 2010). Based on this, cocktails can be constructed using *E. coli* phages that preferentially infect phylogroups B2 and D.

The association of phage infectivity with the phylogenetic group of *E. coli* may be related to the prevalence of phage receptors. Phages initiate infection by binding to host receptors on the bacterial surface. Phage receptors in *E. coli*, which have been reported thus far, include the ferrichrome outer membrane transporter FhuA (Raya et al., 2011), the major outer membrane protein OmpC (Morita et al., 2002), surface glycoconjugates (Kudva et al., 1999), and the O antigen of lipopolysaccharide (LPS) (Perry et al., 2009). Bacteria often develop phage resistance by hindering this critical step of infection using various mechanisms, such as the alteration of phage receptors through spontaneous mutations (Uhl and Miller, 1996), the removal of receptor genes by an insertion sequence (Kim and Ryu, 2011), and the interruption of phage access to host receptors (Kim and Ryu, 2012). The prevalence of host receptors can be related to the phylogenetic group of *E. coli* because the distribution of genes encoding outer membrane proteins, fimbriae, or capsular proteins is different depending on the phylogenetic group (Johnson et al., 2002). Also, the phylogenetic group of *E. coli* is related to the type of the core oligosaccharide of LPS, a common host receptor for phage infection (Amor et al., 2000; Dissanayake et al., 2008). At this stage, further studies are required to examine this possibility.

Phages have been used for the control of foodborne pathogenic bacteria on chicken carcasses. A previous study demonstrated that the treatment with a phage cocktail significantly reduced the number of *Listeria monocytogenes* on chicken carcasses (Cufaoglu and Ayaz, 2019). Atterbury et al. reported that *Campylobacter jejuni* phages effectively infected *C. jejuni* on chicken skin at 4°C (Atterbury et al., 2003). The results in this study showed that phages effectively inhibited *E. coli* on chicken skin particularly at 4°C (Figures 4C, and D). For the control of bacterial contamination of food, temperatures are an important factor affecting the efficacy of phage infection (Seeley and Primrose, 1980; Tokman et al., 2016). The increased efficacy of phage infection at refrigeration temperatures is probably because low temperatures may reduce the emergence of phage-resistant bacteria due to the reduced growth rate of *E. coli* at 4°C compared to 25°C and the limited function of the restriction-modification systems involved in the degradation of phage DNA injected into the host (Dodds et al., 1987; Kim et al., 2012). Additionally, the temperature is a critical environmental factor that determines the viability of phages. Since phages stored at 4°C are more stable than those stored at ≥10°C (Olson et al., 2004), differential phage sensitivity at different temperatures may also affect phage infectivity at 4°C even though the phages were tested at refrigeration temperatures relatively for a short time (24 h) in this study. Regardless of the underlying molecular mechanisms, the increased lytic activity of the phage cocktail on foods (i.e., chicken) at low temperatures may enable the phages to inhibit AMR *E. coli* on raw chicken effectively because raw chicken products are distributed in the cold chain. Given this, the broad host range phage cocktail in this study can be used to control AMR commensal *E. coli* on retail chicken products. To achieve practical application of phages to food, additionally, further studies are needed to develop methods to make phages maintain their infectivity long enough in the food supply chain.

## Data Availability Statement

The datasets presented in this study can be found in online repositories. The names of the repository/repositories and accession number(s) can be found below: https://www.ncbi. nlm.nih.gov/genbank/, MT740314; https://www.ncbi.nlm.nih. gov/genbank/, MT740315; https://www.ncbi.nlm.nih.gov/gen bank/, MT764206; https://www.ncbi.nlm.nih.gov/genbank/, MT764207; and https://www.ncbi.nlm.nih.gov/genbank/, MT764208.

## Author Contributions

JK, SR, and BJ designed the study, analyzed the data, and reviewed the manuscript. JK and HP performed the experiments. JK and BJ wrote the manuscript. All authors contributed to the article and approved the submitted version.

## Conflict of Interest

The authors declare that the research was conducted in the absence of any commercial or financial relationships that could be construed as a potential conflict of interest.

## Publisher’s Note

All claims expressed in this article are solely those of the authors and do not necessarily represent those of their affiliated organizations, or those of the publisher, the editors and the reviewers. Any product that may be evaluated in this article, or claim that may be made by its manufacturer, is not guaranteed or endorsed by the publisher.

## Footnotes

1
https://cge.cbs.dtu.dk/services/ResFinder/

2
https://cge.cbs.dtu.dk/services/VirulenceFinder/

3
https://victor.dsmz.de

4
http://www.ncbi.nlm.nih.gov/nuccore

## References

[B1] Altamirano F. L. G. Barr J. J. (2019). Phage therapy in the postantibiotic era. *Clin. Microbiol. Rev.* 32:e00066–18. 10.1128/cmr.00066-18 30651225 PMC6431132

[B2] Amor K. Heinrichs D. E. Frirdich E. Ziebell K. Johnson R. P. Whitfield C. (2000). Distribution of core oligosaccharide types in lipopolysaccharides from *Escherichia coli*. *Infect. Immun.* 68 1116–1124. 10.1128/iai.68.3.1116-1124.2000 10678915 PMC97256

[B3] Atterbury R. J. Connerton P. L. Dodd C. E. Rees C. E. Connerton I. F. (2003). Application of host-specific bacteriophages to the surface of chicken skin leads to a reduction in recovery of *Campylobacter jejuni*. *Appl. Environ. Microbiol.* 69 6302–6306. 10.1128/aem.69.10.6302-6306.2003 14532096 PMC201188

[B4] Bert F. Johnson J. R. Ouattara B. Leflon-Guibout V. Johnston B. Marcon E. (2010). Genetic diversity and virulence profiles of *Escherichia coli* isolates causing spontaneous bacterial peritonitis and bacteremia in patients with cirrhosis. *J. Clin. Microbiol.* 48 2709–2714. 10.1128/jcm.00516-10 20519468 PMC2916625

[B5] CDC (2013). *Antibiotic Resistance Threats In The United States.* Atlanta, GA: CDC.

[B6] Clermont O. Christenson J. K. Denamur E. Gordon D. M. (2013). The Clermont *Escherichia coli* phylo-typing method revisited: improvement of specificity and detection of new phylo-groups. *Environ. Microbiol. Rep.* 5 58–65. 10.1111/1758-2229.12019 23757131

[B7] Cortés P. Blanc V. Mora A. Dahbi G. Blanco J. E. Blanco M. (2010). Isolation and characterization of potentially pathogenic antimicrobial-resistant *Escherichia coli* strains from chicken and pig farms in Spain. *Appl. Environ. Microbiol.* 76 2799–2805. 10.1128/aem.02421-09 20228098 PMC2863447

[B8] Coura F. M. Diniz S. D. A. Silva M. X. Mussi J. M. S. Barbosa S. M. Lage A. P. (2015). Phylogenetic group determination of *Escherichia coli* isolated from animals samples. *Sci. World J.* 2015 258424–258424. 10.1155/2015/258424 26421310 PMC4572460

[B9] Cufaoglu G. Ayaz N. D. (2019). *Listeria monocytogenes* risk associated with chicken at slaughter and biocontrol with three new bacteriophages. *J. Food Saf.* 39:e12621. 10.1111/jfs.12621

[B10] Dewey-Mattia D. Manikonda K. Hall A. J. Wise M. E. Crowe S. J. (2018). Surveillance for foodborne disease outbreaks—United States, 2009–2015. *MMWR CDC Surveill. Summ.* 67:1. 10.15585/mmwr.ss6710a1 30048426 PMC6061962

[B11] Dissanayake D. Wijewardana T. Gunawardena G. Poxton I. (2008). Distribution of lipopolysaccharide core types among avian pathogenic *Escherichia coli* in relation to the major phylogenetic groups. *Vet. Microbiol.* 132 355–363. 10.1016/j.vetmic.2008.05.024 18597955

[B12] do Monte D. F. Fernandes M. R. Cerdeira L. Esposito F. Galvão J. A. Franco B. D. (2017). Chicken meat as reservoir of colistin-resistant *Escherichia coli* carrying *mcr-1* genes in South America. *Antimicrob. Agents Chemother.* 61:e02718–16. 10.1128/aac.02718-16 28193665 PMC5404526

[B13] Dodds K. L. Perry M. B. Mcdonald I. J. (1987). Alterations in lipopolysaccharide produced by chemostat-grown *Escherichia coli* O157: H7 as a function of growth rate and growth-limiting nutrient. *Can. J. Microbiol.* 33 452–458. 10.1139/m87-075 3300914

[B14] Endersen L. O’mahony J. Hill C. Ross R. P. Mcauliffe O. Coffey A. (2014). Phage therapy in the food industry. *Annu. Rev. Food Sci. Technol.* 5 327–349. 10.1146/annurev-food-030713-092415 24422588

[B15] Farris J. S. (1972). Estimating phylogenetic trees from distance matrices. *Am. Nat.* 106 645–668. 10.1086/282802

[B16] Göker M. García-Blázquez G. Voglmayr H. Tellería M. T. Martín M. P. (2009). Molecular taxonomy of phytopathogenic fungi: a case study in Peronospora. *PLoS One* 4:e6319. 10.1371/journal.pone.0006319 19641601 PMC2712678

[B17] Gu J. Liu X. Li Y. Han W. Lei L. Yang Y. (2012). A method for generation phage cocktail with great therapeutic potential. *PLoS One* 7:e31698. 10.1371/journal.pone.0031698 22396736 PMC3291564

[B18] Johnson J. R. Oswald E. O’bryan T. T. Kuskowski M. A. Spanjaard L. (2002). Phylogenetic distribution of virulence-associated genes among *Escherichia coli* isolates associated with neonatal bacterial meningitis in the Netherlands. *J. Infect. Dis.* 185 774–784. 10.1086/339343 11920295

[B19] Kaper J. B. Nataro J. P. Mobley H. L. (2004). Pathogenic *Escherichia coli*. *Nat. Rev. Microbiol.* 2 123–140. 10.1038/nrmicro818 15040260

[B20] Kim J. Hwang B. K. Choi H. Wang Y. Choi S. H. Ryu S. (2019). Characterization of *mcr-1*-harboring plasmids from pan drug-resistant *Escherichia coli* strains isolated from retail raw chicken in South Korea. *Microorganisms* 7:344. 10.3390/microorganisms7090344 31547260 PMC6780365

[B21] Kim J.-W. Dutta V. Elhanafi D. Lee S. Osborne J. A. Kathariou S. (2012). A novel restriction-modification system is responsible for temperature-dependent phage resistance in *Listeria monocytogenes* ECII. *Appl. Environ. Microbiol.* 78 1995–2004. 10.1128/aem.07086-11 22247158 PMC3298167

[B22] Kim M. Ryu S. (2011). Characterization of a T5-like coliphage, SPC35, and differential development of resistance to SPC35 in *Salmonella enterica* serovar Typhimurium and *Escherichia coli*. *Appl. Environ. Microbiol.* 77 2042–2050. 10.1128/aem.02504-10 21257810 PMC3067339

[B23] Kim M. Ryu S. (2012). Spontaneous and transient defence against bacteriophage by phase-variable glucosylation of O-antigen in *Salmonella enterica* serovar Typhimurium. *Mol. Microbiol.* 86 411–425. 10.1111/j.1365-2958.2012.08202.x 22928771

[B24] King A. M. Lefkowitz E. Adams M. J. Carstens E. B. (2011). *Virus taxonomy: ninth report of the International Committee on Taxonomy of Viruses.* Amsterdam: Elsevier.

[B25] Kluytmans J. A. Overdevest I. T. Willemsen I. Kluytmans-Van Den Bergh M. F. Van Der Zwaluw K. Heck M. (2012). Extended-spectrum β-lactamase–producing *Escherichia coli* from retail chicken meat and humans: comparison of strains, plasmids, resistance genes, and virulence factors. *Clin. Infect. Dis.* 56 478–487. 10.1093/cid/cis929 23243181

[B26] Korf I. H. Meier-Kolthoff J. P. Adriaenssens E. M. Kropinski A. M. Nimtz M. Rohde M. (2019). Still something to discover: novel insights into *Escherichia coli* phage diversity and taxonomy. *Viruses* 11:454. 10.3390/v11050454 31109012 PMC6563267

[B27] Kudva I. T. Jelacic S. Tarr P. I. Youderian P. Hovde C. J. (1999). Biocontrol of *Escherichia coli* O157 with O157-specific bacteriophages. *Appl. Environ. Microbiol.* 65 3767–3773. 10.1128/aem.65.9.3767-3773.1999 10473373 PMC99698

[B28] Labrie S. J. Samson J. E. Moineau S. (2010). Bacteriophage resistance mechanisms. *Nat. Rev. Microbiol.* 8 317–327. 10.1038/nrmicro2315 20348932

[B29] Lefort V. Desper R. Gascuel O. (2015). FastME 2.0: a comprehensive, accurate, and fast distance-based phylogeny inference program. *Mol. Biol. Evol.* 32 2798–2800. 10.1093/molbev/msv150 26130081 PMC4576710

[B30] Liu Y.-Y. Wang Y. Walsh T. R. Yi L.-X. Zhang R. Spencer J. (2016). Emergence of plasmid-mediated colistin resistance mechanism MCR-1 in animals and human beings in China: a microbiological and molecular biological study. *Lancet. Infect. Dis.* 16 161–168. 10.1016/s1473-3099(15)00424-7 26603172

[B31] Meier-Kolthoff J. P. Auch A. F. Klenk H.-P. Göker M. (2013). Genome sequence-based species delimitation with confidence intervals and improved distance functions. *BMC bioinformatics* 14:60. 10.1186/1471-2105-14-60 23432962 PMC3665452

[B32] Meier-Kolthoff J. P. Göker M. (2017). VICTOR: genome-based phylogeny and classification of prokaryotic viruses. *Bioinformatics* 33 3396–3404. 10.1093/bioinformatics/btx440 29036289 PMC5860169

[B33] Meier-Kolthoff J. P. Hahnke R. L. Petersen J. Scheuner C. Michael V. Fiebig A. (2014). Complete genome sequence of DSM 30083 T, the type strain (U5/41 T) of *Escherichia coli*, and a proposal for delineating subspecies in microbial taxonomy. *Stand. Genomic Sci.* 9:2. 10.1186/1944-3277-9-2 25780495 PMC4334874

[B34] Morita M. Tanji Y. Mizoguchi K. Akitsu T. Kijima N. Unno H. (2002). Characterization of a virulent bacteriophage specific for *Escherichia coli* O157:H7 and analysis of its cellular receptor and two tail fiber genes. *FEMS Microbiol. Lett.* 211 77–83. 10.1111/j.1574-6968.2002.tb11206.x 12052554

[B35] Nhung N. T. Chansiripornchai N. Carrique-Mas J. J. (2017). Antimicrobial resistance in bacterial poultry pathogens: a review. *Front. Vet. Sci.* 4:126. 10.3389/fvets.2017.00126 28848739 PMC5554362

[B36] Nilsson A. S. (2014). Phage therapy—constraints and possibilities. *UPS J. Med. Sci.* 119 192–198. 10.3109/03009734.2014.902878 24678769 PMC4034558

[B37] Nogueira T. David P. H. Pothier J. (2019). Antibiotics as both friends and foes of the human gut microbiome: the microbial community approach. *Drug Dev. Res.* 80 86–97. 10.1002/ddr.21466 30370682

[B38] Olson M. R. Axler R. P. Hicks R. E. (2004). Effects of freezing and storage temperature on MS2 viability. *J. Virol. Methods* 122 147–152. 10.1016/j.jviromet.2004.08.010 15542138

[B39] Painter J. A. Hoekstra R. M. Ayers T. Tauxe R. V. Braden C. R. Angulo F. J. (2013). Attribution of foodborne illnesses, hospitalizations, and deaths to food commodities by using outbreak data, United States, 1998-2008. *Emerg. Infect. Dis.* 19 407–415. 10.3201/eid1903.111866 23622497 PMC3647642

[B40] Park H. Kim J. Ryu S. Jeon B. (2019). Predominance of *bla*_CTX*–*M*–*65_ and *bla*_CTX*–*M*–*55_ in extended-spectrum β-lactamase-producing *Escherichia coli* from raw retail chicken in South Korea. *J. Glob. Antimicrob. Resist.* 17 216–220. 10.1016/j.jgar.2019.01.005 30658198

[B41] Perry L. L. Sanmiguel P. Minocha U. Terekhov A. I. Shroyer M. L. Farris L. A. (2009). Sequence analysis of *Escherichia coli* O157:H7 bacteriophage PhiV10 and identification of a phage-encoded immunity protein that modifies the O157 antigen. *FEMS Microbiol. Lett.* 292 182–186. 10.1111/j.1574-6968.2009.01511.x 19210675

[B42] Picard B. Garcia J. S. Gouriou S. Duriez P. Brahimi N. Bingen E. (1999). The link between phylogeny and virulence in *Escherichia coli* extraintestinal infection. *Infect. Immun.* 67 546–553. 10.1128/iai.67.2.546-553.1999 9916057 PMC96353

[B43] Poirel L. Madec J.-Y. Lupo A. Schink A.-K. Kieffer N. Nordmann P. (2018). Antimicrobial resistance in *Escherichia coli*. *Microbiol. Spectr.* 6:4. 10.1128/microbiolspec.ARBA-0026-2017 30003866 PMC11633601

[B44] Rambaut A. (2006). *FigTree 1.4.3 - A Graphical Viewer of Phylogenetic Trees and a Program for Producing Publication-Ready Figures*. Available at: https://mac.softpedia.com/get/Graphics/FigTree.shtml, https://www.ed.ac.uk/edinburgh-infectious-diseases/research/themes/disease-dynamics/andrew-rambaut

[B45] Raya R. R. Oot R. A. Moore-Maley B. Wieland S. Callaway T. R. Kutter E. M. (2011). Naturally resident and exogenously applied T4-like and T5-like bacteriophages can reduce *Escherichia coli* O157:H7 levels in sheep guts. *Bacteriophage* 1 15–24. 10.4161/bact.1.1.14175 21687531 PMC3109454

[B46] Rupp M. E. Fey P. D. (2003). Extended spectrum β-lactamase (ESBL)-producing *Enterobacteriaceae*. *Drugs* 63 353–365. 10.2165/00003495-200363040-00002 12558458

[B47] Saliu E.-M. Vahjen W. Zentek J. (2017). Types and prevalence of extended–spectrum beta–lactamase producing *Enterobacteriaceae* in poultry. *Anim. Health. Res. Rev.* 18 46–57. 10.1017/s1466252317000020 28641596

[B48] Schrauwen E. J. Huizinga P. Van Spreuwel N. Verhulst C. Kluytmans-Van Den Bergh M. F. Kluytmans J. A. (2017). High prevalence of the *mcr-1* gene in retail chicken meat in the Netherlands in 2015. *Antimicrob. Resist. Infect. Control* 6 83. 10.1186/s13756-017-0242-8 28828173 PMC5563067

[B49] Seeley N. Primrose S. (1980). The effect of temperature on the ecology of aquatic bacteriophages. *J. Gen. Virol.* 46 87–95. 10.1099/0022-1317-46-1-87

[B50] Skurnik M. Strauch E. (2006). Phage therapy: facts and fiction. *Int. J. Med. Microbiol.* 296 5–14. 10.1016/j.ijmm.2005.09.002 16423684

[B51] Sulakvelidze A. Alavidze Z. Morris J. G. (2001). Bacteriophage therapy. *Antimicrob. Agents Chemother.* 45 649–659. 10.1128/aac.45.3.649-659.2001 11181338 PMC90351

[B52] Szmolka A. Nagy B. (2013). Multidrug resistant commensal *Escherichia coli* in animals and its impact for public health. *Front. Microbiol.* 4:258. 10.3389/fmicb.2013.00258 24027562 PMC3759790

[B53] Talukdar P. K. Rahman M. Rahman M. Nabi A. Islam Z. Hoque M. M. (2013). Antimicrobial resistance, virulence factors and genetic diversity of *Escherichia coli* isolates from household water supply in Dhaka Bangladesh. *PLoS One* 8:e61090. 10.1371/journal.pone.0061090 23573295 PMC3615999

[B54] Tanji Y. Shimada T. Yoichi M. Miyanaga K. Hori K. Unno H. (2004). Toward rational control of *Escherichia coli* O157:H7 by a phage cocktail. *Appl. Microbiol. Biotechnol.* 64 270–274. 10.1007/s00253-003-1438-9 13680205

[B55] Tokman J. I. Kent D. J. Wiedmann M. Denes T. (2016). Temperature significantly affects the plaquing and adsorption efficiencies of Listeria phages. *Front. Microbiol.* 7:631. 10.3389/fmicb.2016.00631 27199957 PMC4853374

[B56] Uhl M. A. Miller J. F. (1996). Integration of multiple domains in a two-component sensor protein: the *Bordetella pertussis* BvgAS phosphorelay. *EMBO J.* 15 1028–1036. 10.1002/j.1460-2075.1996.tb00440.x 8605872 PMC449998

[B57] Unno T. Han D. Jang J. Lee S.-N. Ko G. Choi H. Y. (2009). Absence of *Escherichia coli* phylogenetic group B2 strains in humans and domesticated animals from Jeonnam Province, Republic of Korea. *Appl. Environ. Microbiol.* 75 5659–5666. 10.1128/aem.00443-09 19592524 PMC2737926

[B58] Wang Y. Zhang R. Li J. Wu Z. Yin W. Schwarz S. (2017). Comprehensive resistome analysis reveals the prevalence of NDM and MCR-1 in Chinese poultry production. *Nat. Microbiol.* 2:16260. 10.21037/jlpm.2017.06.02 28165472

[B59] Xu X. Cui S. Zhang F. Luo Y. Gu Y. Yang B. (2014). Prevalence and characterization of cefotaxime and ciprofloxacin co-resistant *Escherichia coli* isolates in retail chicken carcasses and ground pork. *China. Microb. Drug Resist.* 20 73–81. 10.1089/mdr.2012.0224 23952362

[B60] Yen M. Cairns L. S. Camilli A. (2017). A cocktail of three virulent bacteriophages prevents *Vibrio cholerae* infection in animal models. *Nat. Commun.* 8 1–7. 10.1038/ncomms14187 28146150 PMC5296635

