# Supplementary material for: Inhibition of Antimicrobial-Resistant Escherichia coli Using a Broad Host Range Phage Cocktail Targeting Various Bacterial Phylogenetic Groups
Source: Front Microbiol. 2021 Aug 25;12:699630. doi: 10.3389/fmicb.2021.699630 (PMC8425383; doi:10.3389/fmicb.2021.699630)
Supplement: Supplementary file 1 [file Data_Sheet_1.pdf]

TABLE S1. The phages that were isolated but not included in the cocktail

Phage	Propagation host	Isolation source
JEP2	E35	Sewage
JEP3	E15	Sewage
JEP5	E35	Chicken feces

TABLE S2. Host range of five phages that were included in the phage cocktail

Phylogenetic group	Strain no.[a]	Phage[b]					
		JEP1	JEP4	JEP6	JEP7	JEP8	Cocktail
A	1	I	+++	-	-	-	+++
	2	I	++	-	-	-	++
	3	I	++	++	-	+++	+++
	4	-	-	-	-	++	++
	5	-	+++	-	-	-	+++
	6	I	+	I	++	-	++
	7	I	+	+	+	-	+
	8	-	-	-	-	-	-
	9	++	-	I	++	+++	+++
	10	I	++	++	-	+++	+++
	11	I	+++	++	-	++	+++
	12	+	++	I	++	-	++
	13	-	+++	-	-	-	+++
	14	I	+++	-	-	-	+++
	15	I	+++	-	-	-	+++
	16	-	+	I	I	-	+
	17	-	+++	+++	-	I	+++
	18	-	+++	+++	-	I	+++
	19	-	+++	-	-		+++
	20	I	++	++	-	+++	+++
	21	-	-	I	++	-	++
	22	I	+	I	++	-	++
	23	++	++	I	++	-	++
	24	-	+++	++	I	I	+++
	25	-	+++	I	-	-	+++
	26	I	-	I	I	-	I
	27	-	++	-	-	-	++
	28	-	-	+++	I	-	+++
	29	I	+++	++	-	I	+++
	30	-	+++	-	-	-	+++
	31	I	+++	-	-	-	+++
	32	-	-	-	-	-	-
	33	-	-	I	I	-	I
	34	I	+++	+++	I	-	+++
	35	-	+++	-	-	-	+++
	36	I	+++	-	-	-	+++
	37	-	-	-	++	-	++
	38	-	-	I	-	++	++
B1	39	+++	-	-	++	-	+++
	40	+++	++	+++	+++	-	+++
	41	+++	I	+++	++	-	+++
	42	I	I	+++	-	I	+++
	43	+++	-	-	++	-	+++
	44	+++	-	-	++	-	+++
	45	+++	-	-	++	-	+++
	46	++	-	-	+	-	++
	47	+++	-	-	++	-	+++
	48	+++	-	-	++	+	+++
	49	I	-	-	I	+	+
	50	-	-	-	-	-	-
	51	-	-	-	+	-	+
B2	52	-	-	+++	-	-	+++
	53	I	-	-	-	-	I
	54	-	-	+++	I	-	+++
	55	-	-	+++	I	-	+++

D	56	+++	+++	-	++	-	+++
	57	+++	+++	-	++	-	+++
	58	I	-	++	-	-	++
	59	++	+++	++	++	-	+++
E	60	-	-	+++	I	-	+++
	61	I	-	++	+++	-	+++
	62	-	-	++	+++	-	+++
	63	-	-	-	-	+++	+++
	64	-	-	-	-	+++	+++
	65	-	-	-	-	+++	+++
F	66	-	-	-	I	+++	+++
Unknown	67	I	I	++	-	-	++
**Summary**		**22.4% (15/67)**	**47.8% (32/67)**	**34.3% (23/67)**	**34.3% (23/67)**	**19.4% (13/67)**	**91.0% (61/67)**

a. The 67 strains of AMR E. coli strains were isolated from retail raw chicken.

b. +++, efficiency of plating (EOP) 0.01 to 1; ++, EOP 0.0001 to 0.01; +, EOP < 0.0001; I, bacterial growth inhibition zone; -, no plaque.

TABLE S3. Host range of three phages which were isolated in this study but not included in the phage cocktail

Phylogenetic group	Strain no.[a]	Phage[b]		
		JEP2	JEP3	JEP5
A	1	+++	+++	++
	2	+++	++	++
	3	-	++	-
	4	-	-	-
	5	-	+++	-
	6	-	+	-
	7	-	+	-
	8	-	-	-
	9	-	-	-
	10	-	++	-
	11	-	+++	-
	12	-	++	-
	13	+++	+++	++
	14	-	+++	-
	15	+++	+++	+++
	16	-	+	-
	17	-	+++	-
	18	-	+++	-
	19	+++	+++	++
	20	+++	++	++
	21	-	-	-
	22	-	+	-
	23	-	++	-
	24	-	+++	-
	25	-	+++	-
	26	-	-	-
	27	-	++	-
	28	-	-	-
	29	-	+++	-
	30	+++	+++	+
	31	-	+++	-
	32	-	-	-
	33	-	-	-
	34	-	+++	-
	35	+++	+++	+++
	36	-	+++	-
	37	-	-	-
	38	-	-	-
B1	39	-	-	-
	40	-	++	-
	41	-	I	-
	42	-	I	-
	43	-	-	-
	44	-	-	-
	45	-	-	-
	46	-	-	-
	47	-	-	-
	48	-	-	-
	49	-	-	-
	50	-	-	-
	51	-	-	-
B2	52	-	-	-
	53	-	-	-

	54	-	-	-
	55	-	-	-
D	56	-	+++	-
	57	-	+++	-
	58	-	-	-
	59	-	+++	-
E	60	-	-	-
	61	-	-	-
	62	-	-	-
	63	-	-	-
	64	-	-	-
	65	-	-	-
F	66	-	-	-
Unknown	67	-	I	-
**Summary**		**11.9% (8/67)**	**47.8% (32/67)**	**11.9% (8/67)**

a. The 67 strains of AMR *E. coli* strains were isolated from retail raw chicken.

b. +++, EOP 0.01 to 1; ++, EOP 0.0001 to 0.01; +, EOP < 0.0001; I, bacterial growth inhibition zone; -, no plaque.
